# A Shift towards Pro-Inflammatory CD16+ Monocyte Subsets with Preserved Cytokine Production Potential after Kidney Transplantation

**DOI:** 10.1371/journal.pone.0070152

**Published:** 2013-07-29

**Authors:** Elly J. F. Vereyken, Marina D. Kraaij, Carla C. Baan, Farhad Rezaee, Willem Weimar, Kathryn J. Wood, Pieter J. M. Leenen, Ajda T. Rowshani

**Affiliations:** 1 Department of Nephrology and Transplantation, Erasmus University Medical Center, Rotterdam, The Netherlands; 2 Department of Cell Biology, University Medical Center Groningen, University of Groningen, Groningen, The Netherlands; 3 Transplantation Research Immunology Group, Nuffield Department of Surgical Sciences, University of Oxford, Oxford, United Kingdom; 4 Department of Immunology, Erasmus University Medical Center, Rotterdam, The Netherlands; Agency for Science, Technology and Research - Singapore Immunology Network, Singapore

## Abstract

**Background:**

The presence of monocyte-macrophage lineage cells in rejecting kidney transplants is associated with worse graft outcome. At present, it is still unclear how the monocyte-macrophage related responses develop after transplantation. Here, we studied the dynamics, phenotypic and functional characteristics of circulating monocytes during the first 6 months after transplantation and aimed to establish the differences between kidney transplant recipients and healthy individuals.

**Methods:**

Phenotype, activation status and cytokine production capacity of classical (CD14++CD16−), intermediate (CD14++CD16+) and non-classical (CD14+CD16++), monocytes were determined by flow cytometry in a cohort of 33 healthy individuals, 30 renal transplant recipients at transplantation, 19 recipients at 3 months and 16 recipients at 6 months after transplantation using a cross-sectional approach.

**Results:**

The percentage of both CD16+ monocyte subsets was significantly increased in transplant recipients compared to healthy individuals, indicative of triggered innate immunity (p≤0.039). Enhanced production capacity of tumor necrosis factor-α, interferon-γ and interleukin-1β was observed by monocytes at transplantation compared to healthy individuals. Remarkably, three months post-transplant, in presence of potent immunosuppressive drugs and despite improved kidney function, interferon-γ, tumor necrosis factor-α and interleukin-10 production capacity still remained significantly increased.

**Conclusion:**

Our data demonstrate a skewed balance towards pro-inflammatory CD16+ monocytes that is present at the time of transplantation and retained for at least 6 months after transplantation. This shift could be one of the important drivers of early post-transplant cellular immunity.

## Introduction

Monocyte-macrophage lineage cells next to T-cells are the predominant cell types infiltrating acutely rejecting kidney transplants [1], [2]. The intra-graft presence of macrophages (MΦs) during rejection is associated with worse graft outcome. Although T-cells are known to be required for acute rejection [3]–[5], the degree to which monocytes and MΦs contribute to this process remains incompletely defined.

MΦs are heterogeneous with established roles in tissue injury, homeostasis, remodelling and repair [6], [7]. MΦs can be detected in large numbers in kidney grafts undergoing ischemia/reperfusion injury, during T–cell and antibody–mediated rejection. MΦ infiltration correlated with poor rejection prognosis due to their contribution to early and late inflammatory injury [8], [9]. Depletion of infiltrating MΦs reduced histological features of acute rejection and led to improvement of transplant function in rodent models of kidney transplantation (Tx) [10], [11]. In humans, treatment with vitamin D reduced the number of graft infiltrating MΦs and was associated with increased transplant survival [12]. On the other hand, a protective role was also ascribed to so-called regulatory MΦs [13]. A week prior to kidney Tx regulatory MΦs, which were able to eliminate activated T-cells *in vitro*, were transfused into 2 patients. After 24 weeks these patients needed only low-dose tacrolimus monotherapy to preserve their grafts from rejection [13]. In line, administration of MΦs with specific wound healing and anti-inflammatory phenotypes reduced histological and functional markers of kidney injury in rodents [14], [15].

Monocytes can be subdivided into three phenotypically and functionally distinct subpopulations based on the expression of the lipopolysaccharide (LPS) receptor, CD14, and the Fcγ receptor III, CD16 [16], [17]. In healthy individuals approximately 80–90% of monocytes are highly CD14 positive and CD16 negative (CD14++CD16−): classical monocytes. The remaining 10–20% of monocytes are CD16 positive, which are further subdivided into CD14++CD16+ and CD14+CD16++ cells, intermediate and non-classical monocytes respectively [17]. These monocyte subsets have different chemokine-receptor expression profiles [18]. Important monocytic functions, such as phagocytosis, antigen presentation and cytokine production, are also differently regulated in the monocyte subpopulations [19]–[21]. The monocyte subset composition is altered in several pathologic conditions, including inflammatory and infectious diseases [22] and in coronary heart disease [23]. In kidney transplant recipients, CD14+CD16+ monocytes were associated with subclinical atherosclerosis [24]. In addition, higher numbers of pro-inflammatory CD14+CD16+ monocytes were detected in patients with end-stage renal disease compared to healthy controls [25]–[28]. Monocyte infiltration and specifically glomerular monocytes were associated with graft dysfunction and poor graft outcome [29], [30]. Furthermore, monocytic infiltrates seemed to drive the acute rejection in T-cell-depleted, alemtuzumab-treated kidney transplant recipients [31].

At present, a paucity of data exists regarding the phenotype, dynamics and kinetics of circulating monocytes in relation to Tx and post-transplant complications. We hypothesised that at the time of Tx monocyte subset composition will reflect a higher inflammatory state returning to levels comparable with healthy individuals post-Tx. In this study we determined the dynamics, phenotypic and functional characteristics of peripheral blood monocyte subsets in kidney transplant recipients compared to healthy individuals using a cross-sectional approach.

## Materials and Methods

### Ethics Statement

This study has been approved by the Ethical Committee of the ErasmusMC. All patients have signed written informed consent following the guidelines of the Ethical Committee of the ErasmusMC.

### Patient Characteristics

Whole blood was collected from different groups consisting of 33 healthy individuals (transplant donors), 30 renal recipients at the time of Tx, 19 patients 3 months after Tx, and from 16 patients 6 months after Tx in a cross-sectional approach. All patients were treated homogenously with basiliximab (simulect®) as induction therapy and received triple maintenance immunosuppressive drugs consisting of corticosteroids, calcineurin inhibitors and mycophenolate mofetil according to our local protocol. Corticosteroids (started at 20 mg daily dose) were tapered off to zero at four months after Tx. In addition, the dosing of calcineurin inhibitors was adjusted using the drug trough levels to achieve the pre-defined target trough levels according to protocol. Mycophenolate mofetil was given at a fixed dose. Clinical and immunological characteristics of kidney transplant recipients and kidney donors are listed in Table 1.

**Table 1:** Clinical and immunological characteristics of kidney transplant recipients.

Characteristics	Healthy controlsN = 33	Time of TxN = 30	3 monthspost-Tx N = 19	6 monthspost-Tx N = 16
Age (median (year), range)	51 (25–73)	59 (23–77)	59 (23–69)	62 (23–76)
Gender (% male)	42%	73%	68%	75%
PreTx CMV status (% positive)	48%	53%	58%	43.75%
Dialysis (number of patients, %)		19 (63.3%)	10 (52.6%)	9 (56.3%)
Pre-emptive transplantation		11 (36.7%)	9 (47.4%)	7 (43.8%)
*Transplantation type (%)*				
Living donor, related		11 (36.7%)	8 (42.1%)	7 (43.8%)
Living donor, unrelated		19 (63.3%)	11 (57.9%)	9 (56.3%)
*Primary disease (number of patients, %)*				
Polycystic kidney disease		5 (16.7%)	2 (10,5%)	2 (12.5%)
Diabetic nephropathy		3 (10%)	2 (10.5%)	3 (18.8%)
Hypertension		9 (30%)	8 (42.1%)	6 (37.5%)
IgA nephropathy		2 (6.7%)	2 (10.5%)	1 (6.3%)
Reflux nephropathy		1 (3.3%)	1 (5.3%)	0 (0%)
Glomerulonephritis		3 (10%)	1 (5.3%)	1 (6.3%)
Others		7 (23.3%)	3 (15.8%)	3 (18.8%)
*HLA mismatches total (median, range)*		3 (0–6)	3 (1–5)	3 (2–5)
Class I mismatches		2 (0–4)	2 (0–4)	2 (1–3)
Class II mismatches		1 (0–2)	1 (0–2)	1 (1–2)
Warm ischemia (median (min), range)		19 (11–55)	19 (12–35)	20 (12–35)
*Kidney Function: GFR* (ml/min, median, range)				
GFR at time of Tx		8 (2–21)	7 (2–14)	7 (5–17)
GFR at 3 months postTx		50 (27–82)	55 (26–90)	54.5 (33–76)
GFR at 6 months postTx		47 (19–69)	54 (19–81)	63 (34–81)
*Kidney Function: Serum creatinine* (µmol/l, median, range)				
Creatinine at time of Tx		610.5 (236–1830)	672 (375–1456)	670.5 (319–885)
Creatinine at 3 months postTx		126 (69–379)	122 (46–177)	122 (85–195)
Creatinine at 6 months postTx		137 (91–319)	114 (85–233)	107.5 (69–188)

### Phenotype Characterisation and Activation

To determine the phenotype and activation status of the monocytes, 100 µl whole blood was incubated with conjugated primary antibodies CD3-Amcyan (BD Biosciences), CD3-PE, CD14-Pacfic Blue, CD16-PE-Cy7, CD20-PE, CD56-PE, HLA-DR-APC-Cy7, CD40-FITC, CD80-FITC (all Biolegend), and CD56-APC (eBioscience) for 30 minutes at 4°C. Subsequently the blood was incubated for 10 minutes at room temperature with FACS lysing solution (BD Biosciences) to remove erythrocytes. The cells were washed and analysed using flow cytometry (FACSCanto II, BD Biosciences) and FACSDiva software. Monocytes were characterised based on forward/sideward scatter, lack of expression of CD3, CD20 and CD56 and expression of CD14 and CD16. Conjugated antibodies were used as negative isotype controls. Absolute cell numbers were determined by flow cytometry using Truecount™ tubes (BD Biosciences).

### Intracellular Cytokine Production

Peripheral blood mononuclear cells (PBMCs) were isolated from heparinised blood using Ficoll-Hypaque density gradient (Lymphoprep™). PBMCs were pre-stimulated with 20 ng/ml interferon-gamma (IFN-γ) (U-Cytech) for 2 hours at 37°C followed by overnight treatment with 100 ng/ml LPS (Sigma-Aldrich) and golgiplug (BD Biosciences). After this stimulation ethylenediaminetetraacetic acid was added to the PBMCs for 15 min and cells were washed. Next, extracellular markers, CD3-PE, CD20-PE, CD56-PE, CD14-Pacific Blue and CD16-PE-Cy7, were stained as described above. After treatment with FACS lysing solution, PBMCs were treated with FACS Permeabilizing solution 2 (BD Biosciences) for 15 minutes. Subsequently, conjugated primary antibodies to tumor necrosis factor-alpha (TNF-α)-Percp-Cy5.5, Interleukin-1β (IL-1β)-FITC, IFN-γ-APC-Cy7, IL-6-APC and IL-10-FITC (all Biolegend) were added to determine intracellular cytokine production. The cells were washed and analysed using flow cytometry (FACSCanto II, BD Biosciences) and FACSDiva software. The entire monocyte population was characterised based on forward/sideward scatter, lack of expression of CD3, CD20 and CD56 and expression of CD14 and CD16. Conjugated antibodies were used as negative isotype controls. To exclude the possibility of IFN-γ uptake by monocytes as the reason for increased production capacity of IFN-γ by IFN-γ and LPS-stimulated monocytes, we also used LPS alone to stimulate monocytes.

### Statistical Analysis

Statistical analysis was performed using Kruskal Wallis test and one-way ANOVA with Bonferroni correction in SPSS (15.0.0, Chicago, USA). A p-value <0.05 was considered significant. Data are presented as median±SEM unless otherwise stated.

## Results

### Dynamics of Monocyte Subsets in Kidney Transplant Recipients

**Figure 1:**
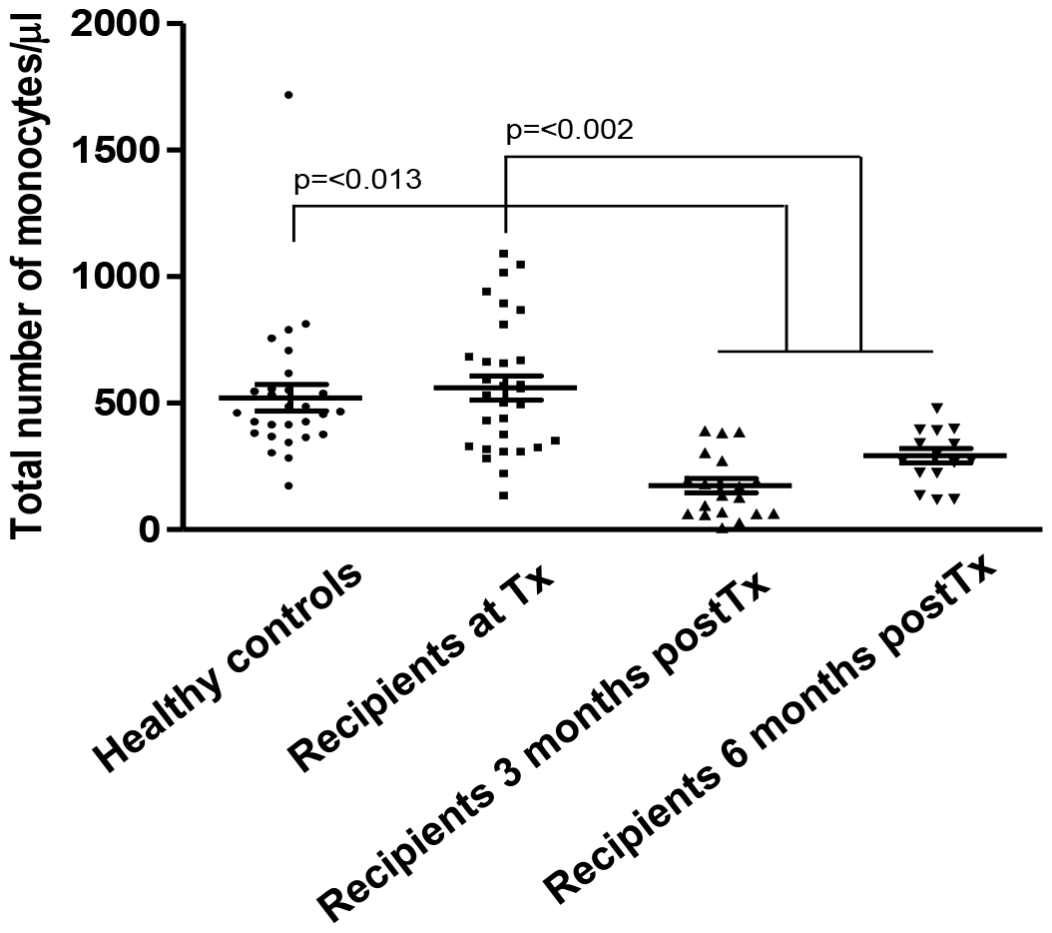
Decreased absolute number of monocytes after kidney transplantation. The absolute number of monocytes is significantly decreased after Tx compared to healthy controls and recipients at the time of Tx. Healthy controls (n = 28), recipient at the time of Tx (n = 30), recipient 3 months post-Tx (n = 19) and 6 months post-Tx (n = 15).

The absolute number of monocytes was similar between transplant recipients at the time of transplantation and healthy controls (Figure 1). The number of monocytes significantly decreased after Tx compared to both healthy controls and recipients at the time of Tx (42±29.5 cells/µl at 3 months and 283±28.9 cells/µl at 6 months vs 538±48.4 cells/µl at the time of Tx and 458±53.5 cells/µl in healthy controls). A trend towards recovery of monocyte number was observed 6 months after Tx.

**Figure 2:**
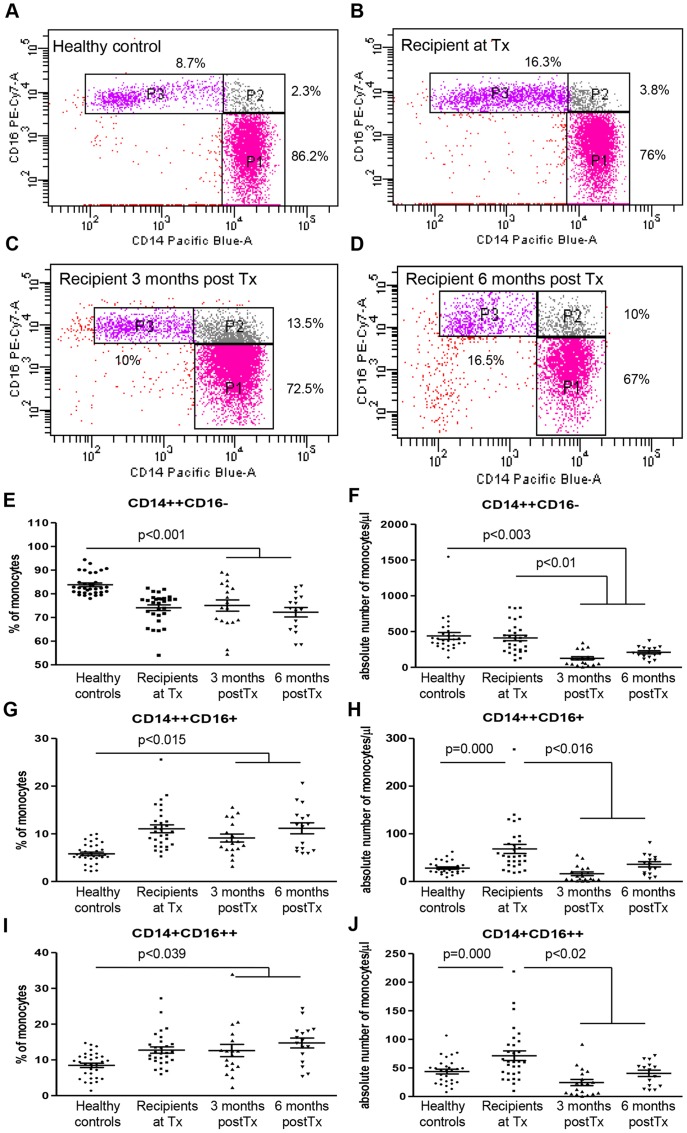
A shift towards CD16+ monocyte subsets in kidney transplant recipients. Representative FACS-plots of CD14/CD16 staining are shown: (A) healthy controls, (B) recipient at the time of Tx, (C) recipient 3 months post-Tx and (D) 6 months post-Tx. The percentage of CD14++CD16− monocytes (E) was significantly decreased at time of Tx in comparison to healthy controls, while the absolute number of CD14++CD16– monocytes (F) remained the same. The increased frequency of CD16+ monocytes, and the concomitant decrease in the classical subset were retained during the post-transplant period. The absolute number of CD14++CD16− monocytes was significantly decreased in the post-transplant period, while the numbers of CD16+ intermediate-non classical monocytes were not altered compared to healthy individuals. The percentage (G and H) and absolute number (I and J) of CD16+ intermediate-non classical monocytes were significantly increased at the time of Tx compared to healthy individuals. Healthy controls (n = 33), recipients at Tx (n = 30), 3 months post-Tx (n = 19), and 6 months post-Tx (n = 15).

CD14 and CD16 cell surface expression revealed that monocyte subset composition was altered at the time of Tx and post-transplant as compared to healthy controls (Figure 2A–D). At the time of Tx the percentage of CD14++CD16− monocytes was significantly decreased in comparison to controls (76.6%±2% vs. 82.4%±0.8%, p = <0.001) (Figure 2E), while the CD16+ monocyte subsets were significantly increased. This rise in CD16+ monocytes was due to a significant increase in both CD14++CD16+ (10.4±2%, vs. 5.4±0.4%, p = <0.001) and CD14+CD16++ subsets (11.8±2%, vs. 8.8±0.6%, p = <0.001) (Figure 2G and I). No difference in absolute number of CD14++CD16− subset could be observed between healthy controls and recipients at time of Tx (377±49.1 vs 380.6±39.4) (Figure 2F). In contrast, absolute numbers of CD14++CD16+ and CD14+CD16++ monocytes were significantly increased compared to controls (53±9.8 and 60.2±8.5 vs 25.5±2.5 and 44.5±4.3) (Figure 2H and J).

Surprisingly, even under potent triple immunosuppressive therapy and despite recovery of kidney function after Tx (Table 1), sustained quantitative and qualitative changes in monocyte subsets remained present, distinguishing transplant population from healthy individuals. Both 3 and 6 months after Tx the percentage of CD14++CD16− monocytes was significantly lower compared to healthy controls (74.2±2.4% and 73.6±2.1% vs 82.4%±0.8%, p = <0.001), while a sustained significant rise in the percentage of CD16+ monocytes was observed at both time points post-Tx (Figure 2G and I, p = <0.001). Again both CD14++CD16+ and CD14+CD16++ monocyte subsets contributed to this significant rise in frequency (8.9±0.9% and 11.9±1.2% at 3 months, 11.8±1.2% and 15.2±1.2% at 6 months vs 5.4±0.4% and 8.8±0.6% respectively, p = <0.039). In our cohort, the total number of monocytes was decreased after Tx in comparison to healthy individuals. The number of CD14++CD16− monocytes was significantly lower at post-Tx time points compared to healthy controls (121.6±23.5 at 3 months and 195.6±22.7 at 6 months vs 377.4±49.1, p = <0.003). However, the number of CD16+ monocytes, both CD14++CD16+ (6.8±3.9 at 3 months and 37.1±5.6 at 6 months vs 25.5±2.5) and CD14+CD16++ (22.3±5.6 at 3 months and 47.5±5.7 at 6 months vs 44.5±4.3) subsets, remained constant after Tx and were comparable to those found in healthy controls (Figure 2H and J).

Thus, although the absolute number of monocytes decreased in the post-transplant period, subset distribution remained constant and similar to the skewed distribution of monocyte subsets present at the time of Tx, with a relative over-representation of CD16+ monocytes.

### Expression of Co-stimulatory Molecules by Monocyte Subsets in Kidney Transplant Recipients

**Figure 3:**
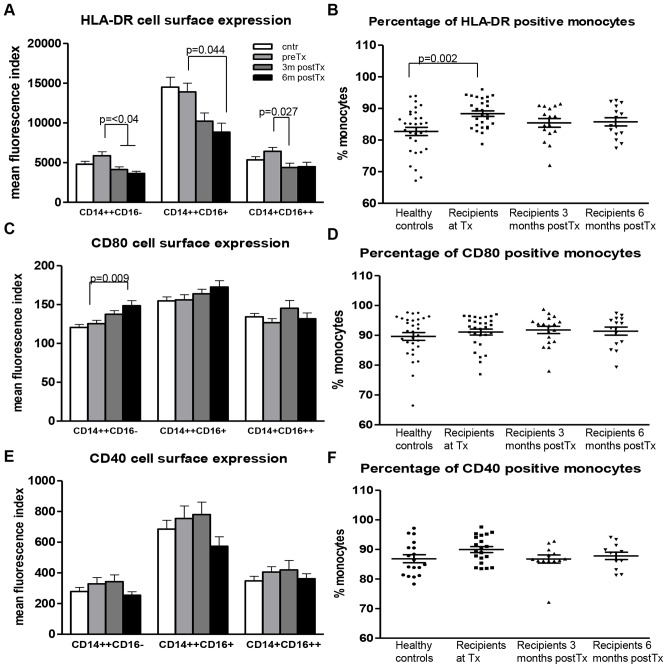
Expression of co-stimulatory molecules by monocytes in kidney transplant recipients. (A) HLA-DR expression was decreased after Tx compared to recipients at the time of Tx. (B) The percentage of HLA-DR positive monocytes was significantly increased at the time of Tx compared to healthy controls. CD80 expression (C) and the percentage of CD80 positive monocytes (D) did not differ between recipients at time of Tx and healthy controls. The CD40 expression (E) and the percentage of CD40 expressing monocytes (F) were similar in all the groups tested. Healthy controls (n = 33), recipients at Tx (n = 30), 3 months post-Tx (n = 19), and 6 months post-Tx (n = 16).

In order to determine the activation status of circulating monocytes, cell surface expression of HLA-DR and co-stimulatory molecules, CD80 and CD40 was measured. In all groups HLA-DR cell surface expression, as measured by mean fluorescence index (MFI), was significantly higher in CD14++CD16+ monocytes compared to CD14++CD16− and CD14+CD16++ (Figure 3A, p = <0.002). The level of HLA-DR cell surface expression was comparable between healthy donors and recipients at the time of Tx (Figure 3A). In contrast, the percentage of HLA-DR positive monocytes was significantly higher in patients at the time of Tx compared to healthy controls (Figure 3B, p = 0.002). Interestingly, after Tx a decrease in HLA-DR cell surface expression level was observed in all monocyte subsets compared to the time of Tx, reaching significance in classical and non-classical monocytes at 3 months compared to the time of Tx, and in the classical and intermediate monocyte subsets at 6 months after Tx.

The pattern of CD80 expression was similar in all groups tested, with a significantly higher expression of CD80 in CD14++CD16+ monocytes compared to CD14++CD16− and CD14+CD16++ (Figure 3C, p = <0.05). Both CD80 cell surface expression level, as measured by MFI, and the percentage of CD80 positive monocytes did not differ between transplant recipients at time of Tx and healthy controls (Figure 3C and D). A trend towards higher CD80 cell surface expression was seen during the post-transplant period compared to the time of Tx reaching statistical significance in CD14++CD16− monocytes 6 months after Tx (p = 0.009).

Similar to CD80, no difference was observed in both percentage of CD40 positive monocytes and CD40 cell surface expression between healthy controls and kidney transplant recipients in all different subsets tested (Figure 3E and F). The cell surface expression level of CD40, displayed a trend towards higher expression at the time of Tx and 3 months thereafter compared to healthy controls. At 6 months, CD40 expression was decreased but still remained comparable to healthy controls.

### Intracellular Cytokine Production by Monocytes in Kidney Transplant Recipients

**Figure 4:**
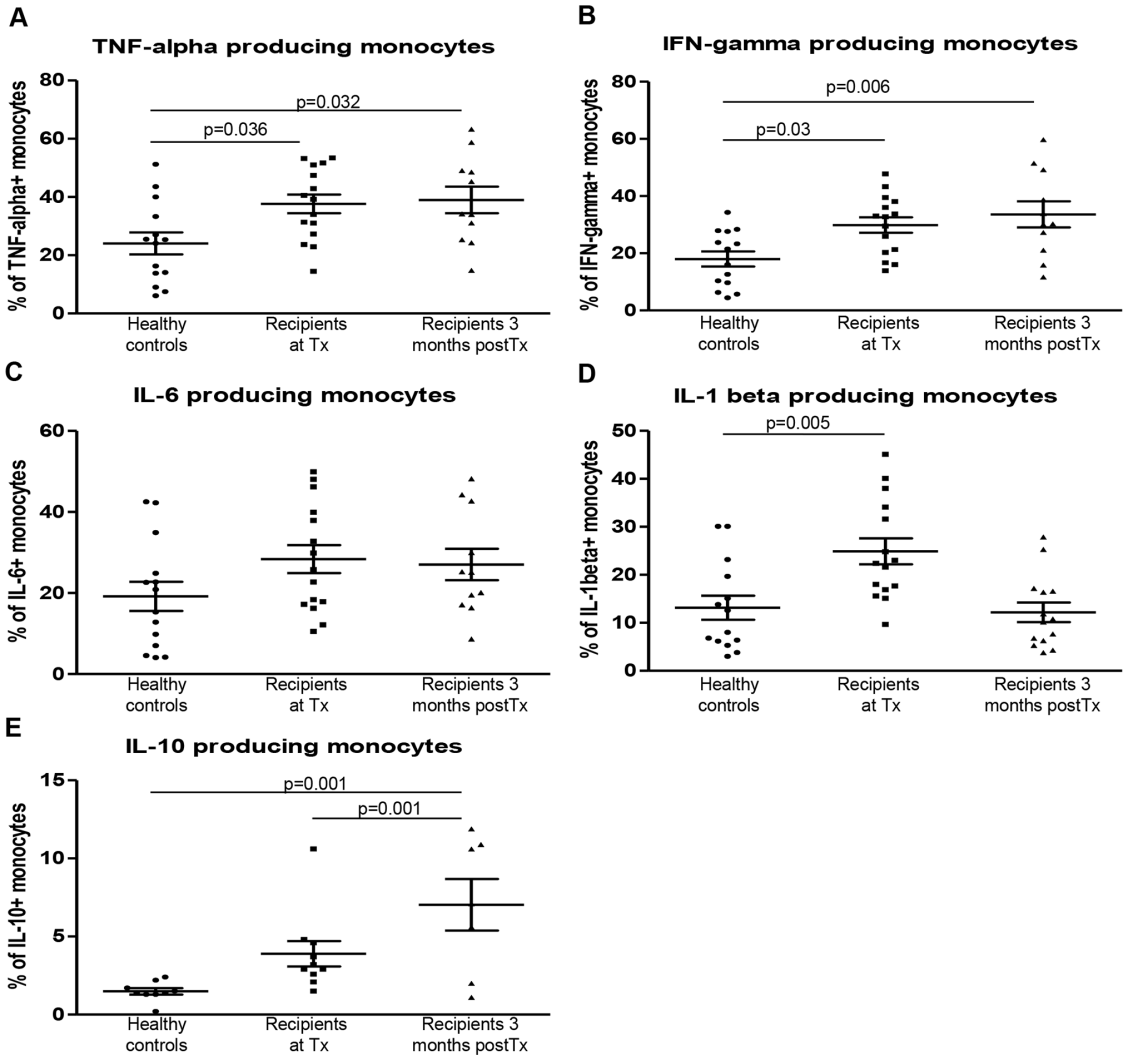
Production of cytokines by monocytes in kidney transplant recipients. Production of cytokines was tested after stimulation of freshly isolated PBMCs of healthy controls, recipients at the time of Tx and recipients at 3 months after Tx with IFN-γ and LPS in the presence of golgiplug. The entire monocyte population was determined based on forward/sideward scatter, lack of expression of CD3, CD20 and CD56 and expression of CD14 and CD16. The percentage of (A) TNF-α and (B) IFN-γ producing monocytes was significantly higher in patients both at the time of Tx and at 3 months after Tx compared to healthy controls (p = <0.03). (C) IL-6 production was not different between the groups tested. (D) The production of IL-1β was significantly increased in patients at the time of Tx compared to healthy controls. (E) The percentage of IL-10 producing monocytes was significantly higher 3 months after Tx compared to both healthy controls and recipients at the time of Tx. Healthy controls (n = 14; n = 9 for IL-10), recipients at Tx (n = 15; n = 10 for IL-10), and 3 months post-Tx (n = 11; n = 7 for IL-10).

Next, we hypothesized that the capacity of monocytes to produce pro- and anti- inflammatory cytokines would be affected by post-transplant early immunity and the use of immunosuppressive drugs. Therefore we measured the production of pro-inflammatory cytokines TNF-α, IFN-γ, IL-6 and IL-1β, and anti-inflammatory cytokine IL-10 by monocytes obtained at the time of Tx and at 3 months post-transplant (Figure 4). Representative FACS plots of unstimulated and stimulated monocytes and the isotype controls are depicted in Figures S1–7.

The percentage of TNF-α producing monocytes was significantly increased in patients at the time of Tx compared to healthy controls (39.2%±3.3 vs. 24.8%±3.9, p = 0.036) (Figure 4A). Remarkably, despite recovery of kidney function and use of immunosuppressive drugs 3 months after Tx, the percentage of TNF-α producing monocytes still remained significantly higher in comparison to healthy controls (34.2%±3.8 vs. 24.8%±3.9, p = 0.032). To our surprise, kidney transplant recipients had a significantly higher percentage of IFN-γ producing monocytes not only at the time of Tx but also 3 months thereafter (32.8%±2.8 vs. 18.8%±2.7 p = 0.03 and 30.3%±3.9 vs. 18.8%±2.7 p = 0.006) (Figure 4B). This indicates a higher potential of monocytes to produce dominant pro-inflammatory cytokines that is retained during the first 3 months after Tx and not diminished by immunosuppression. Stimulation with LPS alone also significantly increased the percentage of IFN-γ positive monocytes, indicating that the increased IFN-γ production capacity by monocytes was not due to uptake from the supernatant (Figure S8).

The production of IL-6 did not differ significantly between recipients and healthy controls at the time points measured (Figure 4C). A significantly higher percentage of monocytes produced IL-1β in recipients at the time of Tx compared to healthy controls (22.4%±2.8 vs.10.3%±2.6, p = 0.005) (Figure 4D). Three months after Tx the percentage of IL-1β producing monocytes was back to healthy control levels (11.9%±2.3 vs 10.3%±2.6). Although a bimodal distribution could be observed in IL-10 producing monocytes after Tx, which might be attributable to the limited numbers tested, the percentage IL-10 producing monocytes was significantly higher 3 months post-Tx compared to both healthy controls and recipients at the time of Tx (7.1%±1.4 vs 1.4%±0.2 and 3.1%±0.7, p = 0.001) (Figure 4E). Importantly, no difference in percentage of IL-10 producing monocytes was observed between recipients at the time of Tx and healthy controls, indicating their preserved IL-10 producing capacity.

## Discussion

Our study reveals that the monocyte subset composition is significantly altered in kidney transplant recipients as compared to healthy individuals at pre- and post-Tx time points. The balance is skewed towards the pro-inflammatory intermediate and non-classical subsets for at least six months after Tx even though the total number of monocytes is decreased. At Tx, the monocytes possess the potential to produce significantly higher levels of dominant pro-inflammatory cytokines IL-1β, TNF-α and IFN-γ. Even in the presence of potent triple immunosuppression, decreased total number of monocytes and despite recovered kidney function, the cytokine production capacity of monocytes remains higher than the healthy control group during the examined post-Tx period. This shift in dynamics and characteristics of monocyte subsets could be one of the important cellular drivers of early post-transplant immunity.

In line with our results, the presence of an increased percentage of intermediate and non-classical monocytes in end-stage kidney disease patients undergoing dialysis has been reported [25], [26], [28], [32], [33] Scherberich et al. found an increase in the monocyte subset co-expressing CD14 with CD16 in patients with chronic renal failure [27]. The authors investigated the effect of different immunosuppressive regimens on the frequency of intermediate and non-classical monocytes. No difference was observed between the different medication groups [27]. This is notable, since glucocorticoids are cornerstone drugs used after Tx and a preferential decrease of the CD14+CD16+ monocyte population by glucocorticoids has been described [34]. Ulrich et al. showed a significant decrease in the percentage of CD14+CD16+ monocytes in kidney transplant recipients receiving methylprednisolone in combination with other immunosuppressive drugs compared to haemodialysis patients [24]. We could not confirm steroid-induced effects in our population, as patients were treated homogeneously with prednisone up to 4 months after Tx. Differences in dosing or relative overrepresentation of intermediate and non-classical monocytes might explain these different observations despite their higher sensitivity for corticosteroids.

We measured monocyte activation status based on the expression of co-stimulatory molecules. Compared to healthy controls, the percentage of HLA-DR positive monocytes was higher at the time of Tx indicating a triggered activation status. Surprisingly, HLA-DR and CD40 cell surface expression in the post-transplant period was comparable to healthy controls, indicating that immunosuppression did not lower the activation status of monocytes beyond healthy control levels. A trend towards higher CD80 expression was visible in the post-transplant period, which might imply a higher pro-inflammatory set-point compared to healthy controls.

Even though the absolute number of monocytes was decreased after Tx, the percentage of TNF-α and IFN-γ producing monocytes was significantly increased in kidney transplant recipients at the time of Tx and at 3 months post-transplant compared to healthy controls. Both stimulation with IFN-γ and LPS or LPS alone resulted in an increase in the percentage of IFN-γ producing monocytes, which is a re-confirmation of the presence of the higher production capacity of IFN-γ by monocytes in our population of interest. Even though the CD14 staining patterns are somewhat distorted, probably due to the stimulation, apoptosis and/or formed clumps, it is clear that the cytokine production is enhanced after stimulation. We also demonstrated that IFN-γ can be produced by monocytes obtained from kidney transplant recipients using stimulation protocols (IFN-γ and LPS or LPS alone) at both mRNA and protein level (unpublished data). Although IFN-γ production by human monocyte-derived MΦs and dendritic cells were described previously [35]–[37], a recently published report demonstrated production of high IFN-γ levels by human monocytes in the presence of IL-2 and the bisphosphonate zoledronic acid [38]. These data are in line with our finding that human monocytes can produce IFN-γ. Also the percentage of IL-1β positive monocytes was found to be significantly higher at the time of Tx. In contrast to TNF-α and IFN-γ however, 3 months after Tx the percentage of IL-1β positive monocytes was back to healthy control levels. The increase in the percentage of monocytes producing pro-inflammatory cytokines could be related to the increased percentage of intermediate and non-classical monocytes. These monocytes are believed to be the main producers of TNF-α as shown by the reduction in TNF-α production after depletion of CD16+ monocytes *in vitro* [19], although the possibility that different monocyte subsets in transplant recipients may behave differently from those in healthy controls upon activation cannot be ruled out.

TNF-α, and IFN-γ are dominant pro-inflammatory cytokines involved in transplant rejection [39], [40]. De Serres et al. demonstrated that secretion of pro-inflammatory cytokines IL-1β, IL-6 and TNF-α by circulating monocytes is associated with transplant glomerulopathy in kidney transplant recipients, indicating a pivotal role for monocytes in chronic post-transplant inflammation [41]. Furthermore, monocytes of liver transplant recipients who experienced rejection showed an enhanced capacity to produce TNF-α and IL-6 compared to patients who did not develop rejection [42]. IL-6 was shown to be a main driver of chronic cardiac allograft dysfunction [43]. In murine transplant models, high IFN-γ gene expression levels were associated with rejection [44] and IFN-γ−/− mice were incapable of rejecting MHCII incompatible grafts [45]. In humans, high pre-transplant IFN-γ plasma levels and IFN-γ production during a mixed lymphocyte reaction were associated with acute rejection episodes [46], [47] and a predictor of long term graft function [48]. IFN-γ mRNA was significantly higher in patients with pronounced clinical glomerulitis compared to patients with subclinical glomerulitis and patients without any histological abnormalities [49].

Surprisingly, the percentage of IL-10-producing monocytes appeared to be significantly increased at 3 months post-Tx, although the results indicate that not all recipients have an increased IL-10 producing capability. This increase in IL-10 production in combination with the increased expression of pro-inflammatory cytokines might be attributed to a triggered innate immune system. Accordingly, Cartwright et al. observed that high production of IL-10 in a mixed lymphocyte reaction pre-Tx was strongly associated with rejection [46].

In conclusion, our data point to a prolonged shift towards pro-inflammatory intermediate and non-classical monocyte subsets in kidney transplant recipients, paralleled by an increased potential of cytokine production, despite recovered kidney function and the use of potent immunosuppressive drugs. The question arises whether skewing of the innate immune system towards a more pro-inflammatory set-point facilitates the occurrence of acute and/or chronic rejection. Further studies are needed to elucidate the functional implications of these alterations.

## Supporting Information

Figure S1 **TNF-α producing monocytes in kidney transplant recipients.** Production of TNF-α was tested after no stimulation or combined stimulation of freshly isolated PBMCs of healthy controls (A and B), recipients at the time of Tx (D and E) and recipients at 3 months after Tx (G and H) with IFN-γ and LPS in the presence of golgiplug. (C, F, I) Corresponding histograms for unstimulated (dashed line) and stimulated (solid line) cells. The monocyte population was determined based on forward/sideward scatter, lack of expression of CD3, CD20 and CD56 and expression of CD14 and CD16. Representative FACS plots of intracellular cytokine production are shown.
(TIF)

Figure S2 **IFN-γ producing monocytes in kidney transplant recipients.** Production of IFN-γ was tested after no stimulation or combined stimulation of freshly isolated PBMCs of healthy controls (A and B), recipients at the time of Tx (D and E) and recipients at 3 months after Tx (G and H) with IFN-γ and LPS in the presence of golgiplug. (C, F, I) Corresponding histograms for unstimulated (dashed line) and stimulated (solid line) cells. The monocyte population was determined based on forward/sideward scatter, lack of expression of CD3, CD20 and CD56 and expression of CD14 and CD16. Representative FACS plots of intracellular cytokine production are shown.
(TIF)

Figure S3 **IL-6 producing monocytes in kidney transplant recipients.** Production of IL-6 was tested after no stimulation or combined stimulation of freshly isolated PBMCs of healthy controls (A and B), recipients at the time of Tx (D and E) and recipients at 3 months after Tx (G and H) with IFN-γ and LPS in the presence of golgiplug. (C, F, I) Corresponding histograms for unstimulated (dashed line) and stimulated (solid line) cells. The monocyte population was determined based on forward/sideward scatter, lack of expression of CD3, CD20 and CD56 and expression of CD14 and CD16. Representative FACS plots of intracellular cytokine production are shown.
(TIF)

Figure S4 **IL-1β producing monocytes in kidney transplant recipients.** Production of IL-1β was tested after no stimulation or combined stimulation of freshly isolated PBMCs of healthy controls (A and B), recipients at the time of Tx (D and E) and recipients at 3 months after Tx (G and H) with IFN-γ and LPS in the presence of golgiplug. (C, F, I) Corresponding histograms for unstimulated (dashed line) and stimulated (solid line) cells. The monocyte population was determined based on forward/sideward scatter, lack of expression of CD3, CD20 and CD56 and expression of CD14 and CD16. Representative FACS plots of intracellular cytokine production are shown.
(TIF)

Figure S5 **IL-10 producing monocytes in kidney transplant recipients.** Production of IL-10 was tested after no stimulation or combined stimulation of freshly isolated PBMCs of healthy controls (A and B), recipients at the time of Tx (D and E) and recipients at 3 months after Tx (G and H) with IFN-γ and LPS in the presence of golgiplug. (C, F, I) Corresponding histograms for unstimulated (dashed line) and stimulated (solid line) cells. The monocyte population was determined based on forward/sideward scatter, lack of expression of CD3, CD20 and CD56 and expression of CD14 and CD16. Representative FACS plots of intracellular cytokine production are shown.
(TIF)

Figure S6 **Isotype controls in unstimulated cells obtained from kidney transplant recipients.** Representative FACS plots are shown of the isotype controls for the intracellular cytokine staining. Isotype controls in unstimulated cells for TNF-α, IFN-γ, IL-6, IL-1β and IL-10 (left side) and cytokine staining in unstimulated cells for TNF-α, IFN-γ, IL-6, IL-1β and IL-10 (right side).
(TIF)

Figure S7 **Isotype controls in IFN-γ and LPS stimulated cells obtained from kidney transplant recipients.** Representative FACS plots are shown of the isotype controls for the intracellular cytokine staining. Isotype controls in IFN-γ and LPS stimulated cells for TNF-α, IFN-γ, IL-6, IL-1β and IL-10 (left side) and cytokine staining in IFN-γ and LPS stimulated cells for TNF-α, IFN-γ, IL-6, IL-1β and IL-10 (right side).
(TIF)

Figure S8 **Percentage of IFN-γ producing monocytes after stimulation with either IFN-γ and LPS or LPS alone.** Production of IFN-γ was tested after stimulation of freshly isolated PBMCs from recipients at time of Tx with either the combination of IFN-γ and LPS or LPS alone in the presence of golgiplug. The monocyte population was determined based on forward/sideward scatter, lack of expression of CD3, CD20 and CD56 and expression of CD14 and CD16. (A) Histogram of IFN-γ production of unstimulated monocytes (dashed line), IFN-γ and LPS stimulated (solid line) and LPS stimulated (dotted line). (B) The ratio of the percentage of IFN-γ producing monocytes with IFN-γ and LPS or LPS stimulation alone compared to the unstimulated situation was comparable.
(TIF)
