# Supplementary figures and images for: A Shift towards Pro-Inflammatory CD16+ Monocyte Subsets with Preserved Cytokine Production Potential after Kidney Transplantation
Source: PLoS One. 2013 Jul 29;8(7):e70152. doi: 10.1371/journal.pone.0070152 (PMC3726371; doi:10.1371/journal.pone.0070152)

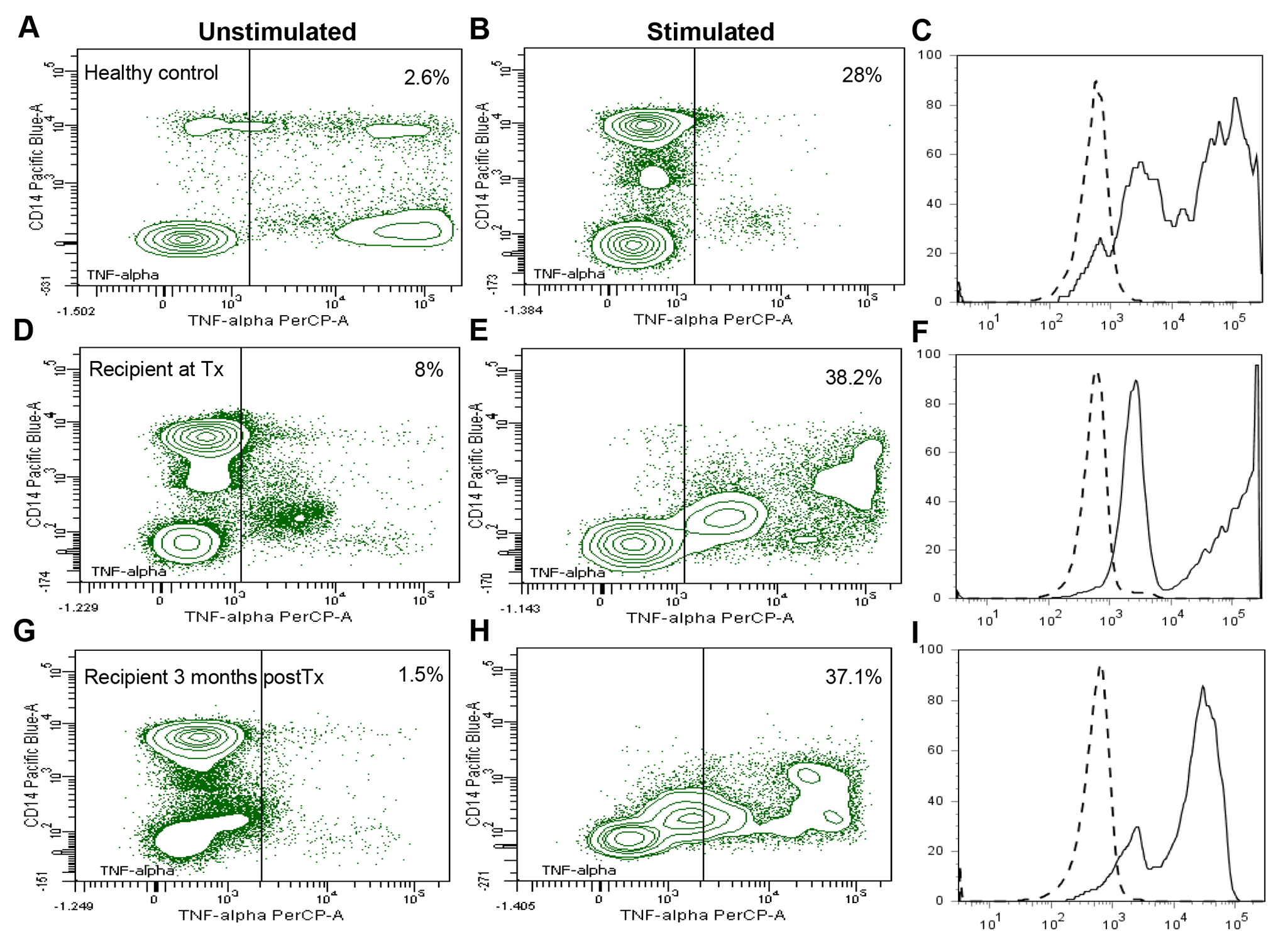

Supplement: Figure S1 — TNF-α producing monocytes in kidney transplant recipients. Production of TNF-α was tested after no stimulation or combined stimulation of freshly isolated PBMCs of healthy controls (A and B), recipients at the time of Tx (D and E) and recipients at 3 months after Tx (G and H) with IFN-γ and LPS in the presence of golgiplug. (C, F, I) Corresponding histograms for unstimulated (dashed line) and stimulated (solid line) cells. The monocyte population was determined based on forward/sideward scatter, lack of expression of CD3, CD20 and CD56 and expression of CD14 and CD16. Representative FACS plots of intracellular cytokine production are shown. (TIF) [file pone.0070152.s001.tif]

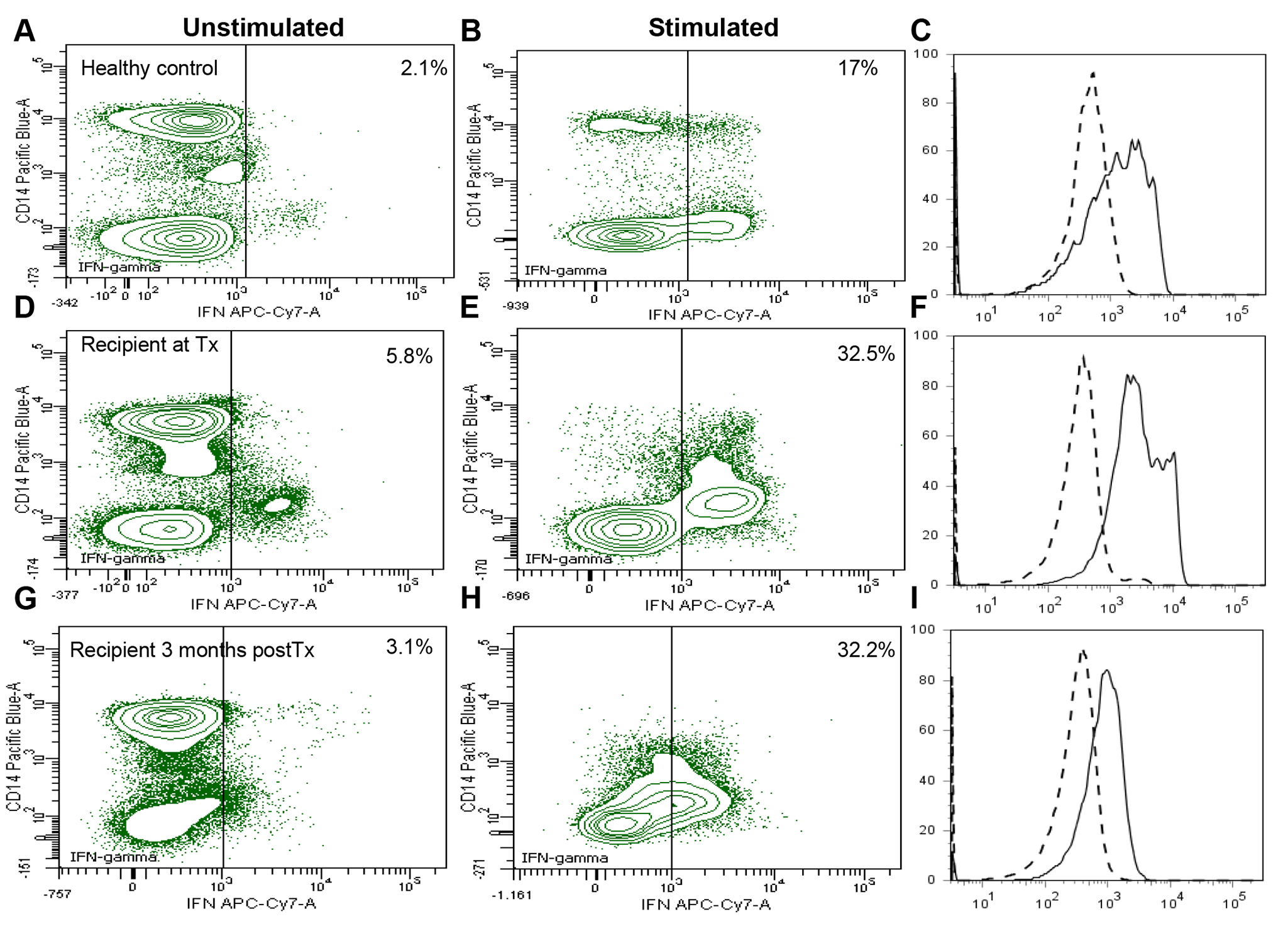

Supplement: Figure S2 — IFN-γ producing monocytes in kidney transplant recipients. Production of IFN-γ was tested after no stimulation or combined stimulation of freshly isolated PBMCs of healthy controls (A and B), recipients at the time of Tx (D and E) and recipients at 3 months after Tx (G and H) with IFN-γ and LPS in the presence of golgiplug. (C, F, I) Corresponding histograms for unstimulated (dashed line) and stimulated (solid line) cells. The monocyte population was determined based on forward/sideward scatter, lack of expression of CD3, CD20 and CD56 and expression of CD14 and CD16. Representative FACS plots of intracellular cytokine production are shown. (TIF) [file pone.0070152.s002.tif]

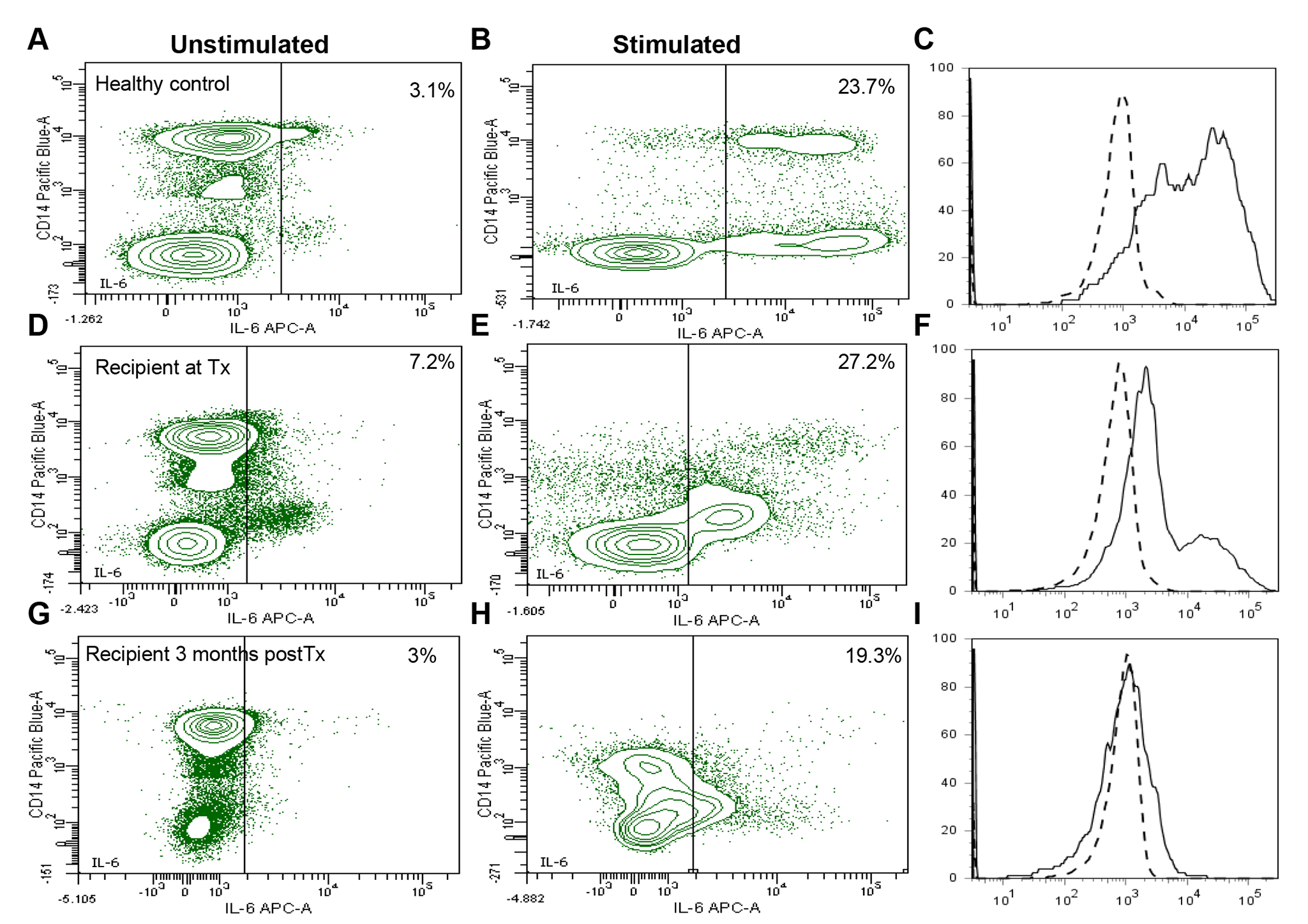

Supplement: Figure S3 — IL-6 producing monocytes in kidney transplant recipients. Production of IL-6 was tested after no stimulation or combined stimulation of freshly isolated PBMCs of healthy controls (A and B), recipients at the time of Tx (D and E) and recipients at 3 months after Tx (G and H) with IFN-γ and LPS in the presence of golgiplug. (C, F, I) Corresponding histograms for unstimulated (dashed line) and stimulated (solid line) cells. The monocyte population was determined based on forward/sideward scatter, lack of expression of CD3, CD20 and CD56 and expression of CD14 and CD16. Representative FACS plots of intracellular cytokine production are shown. (TIF) [file pone.0070152.s003.tif]

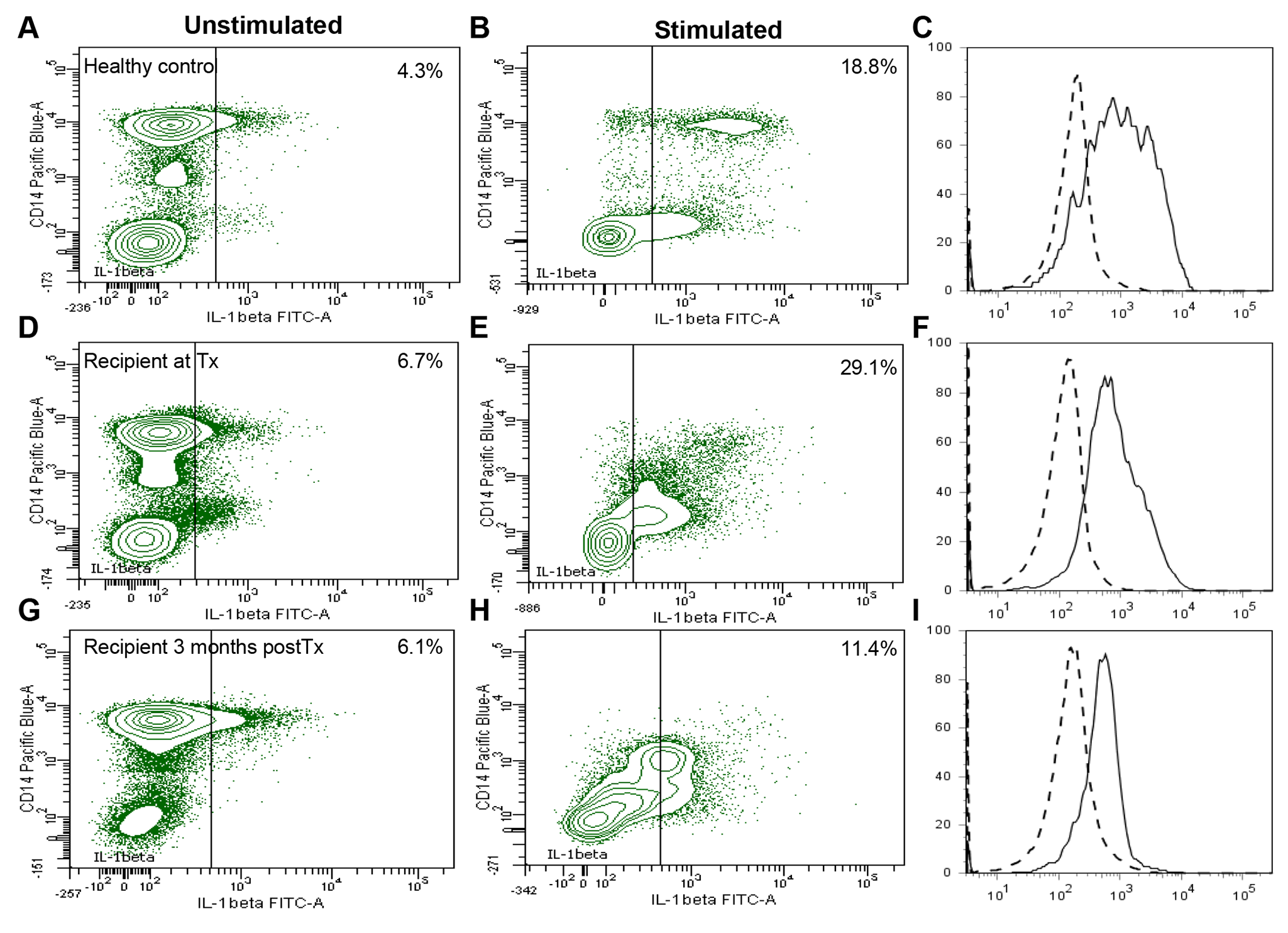

Supplement: Figure S4 — IL-1β producing monocytes in kidney transplant recipients. Production of IL-1β was tested after no stimulation or combined stimulation of freshly isolated PBMCs of healthy controls (A and B), recipients at the time of Tx (D and E) and recipients at 3 months after Tx (G and H) with IFN-γ and LPS in the presence of golgiplug. (C, F, I) Corresponding histograms for unstimulated (dashed line) and stimulated (solid line) cells. The monocyte population was determined based on forward/sideward scatter, lack of expression of CD3, CD20 and CD56 and expression of CD14 and CD16. Representative FACS plots of intracellular cytokine production are shown. (TIF) [file pone.0070152.s004.tif]

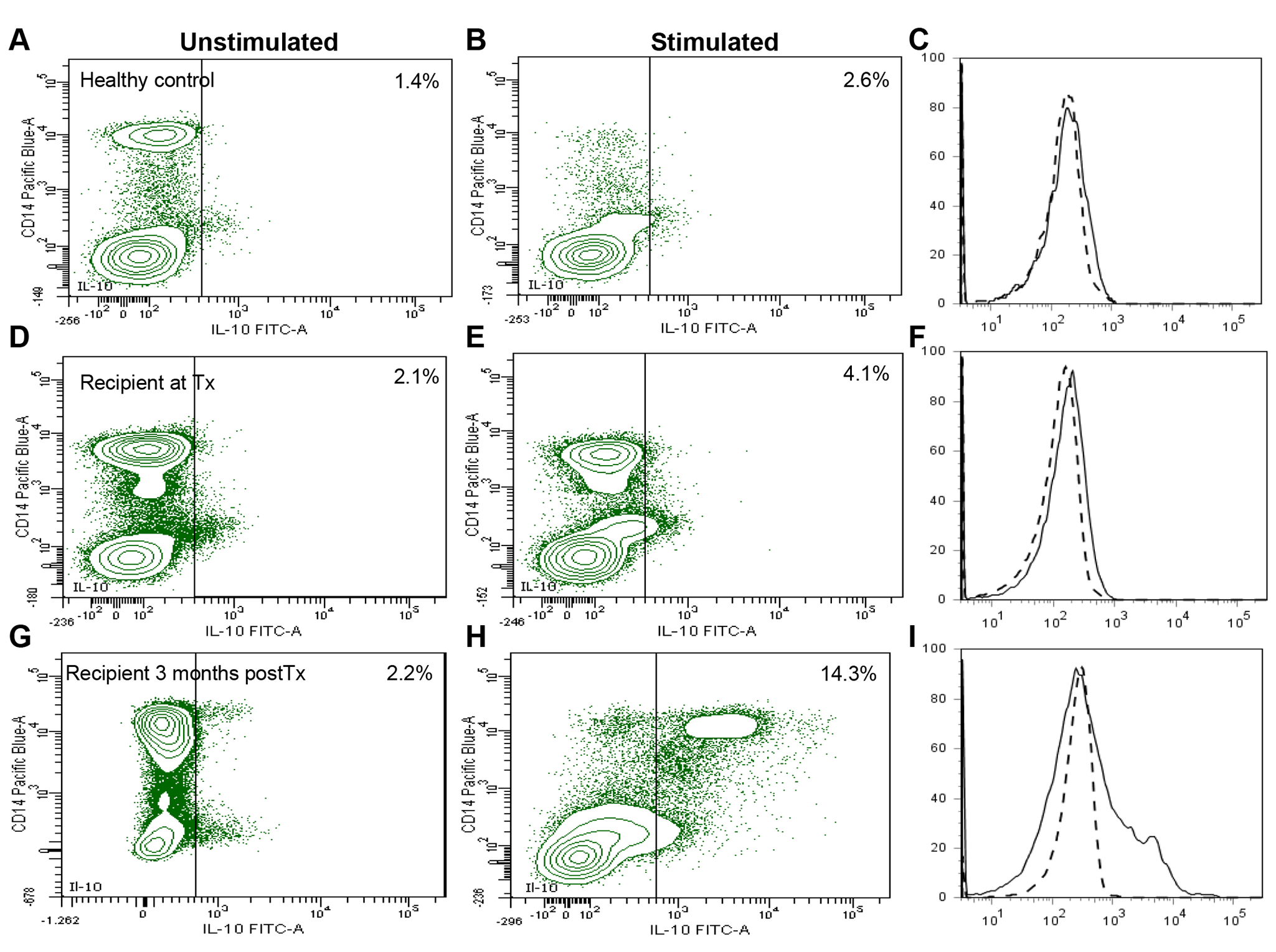

Supplement: Figure S5 — IL-10 producing monocytes in kidney transplant recipients. Production of IL-10 was tested after no stimulation or combined stimulation of freshly isolated PBMCs of healthy controls (A and B), recipients at the time of Tx (D and E) and recipients at 3 months after Tx (G and H) with IFN-γ and LPS in the presence of golgiplug. (C, F, I) Corresponding histograms for unstimulated (dashed line) and stimulated (solid line) cells. The monocyte population was determined based on forward/sideward scatter, lack of expression of CD3, CD20 and CD56 and expression of CD14 and CD16. Representative FACS plots of intracellular cytokine production are shown. (TIF) [file pone.0070152.s005.tif]

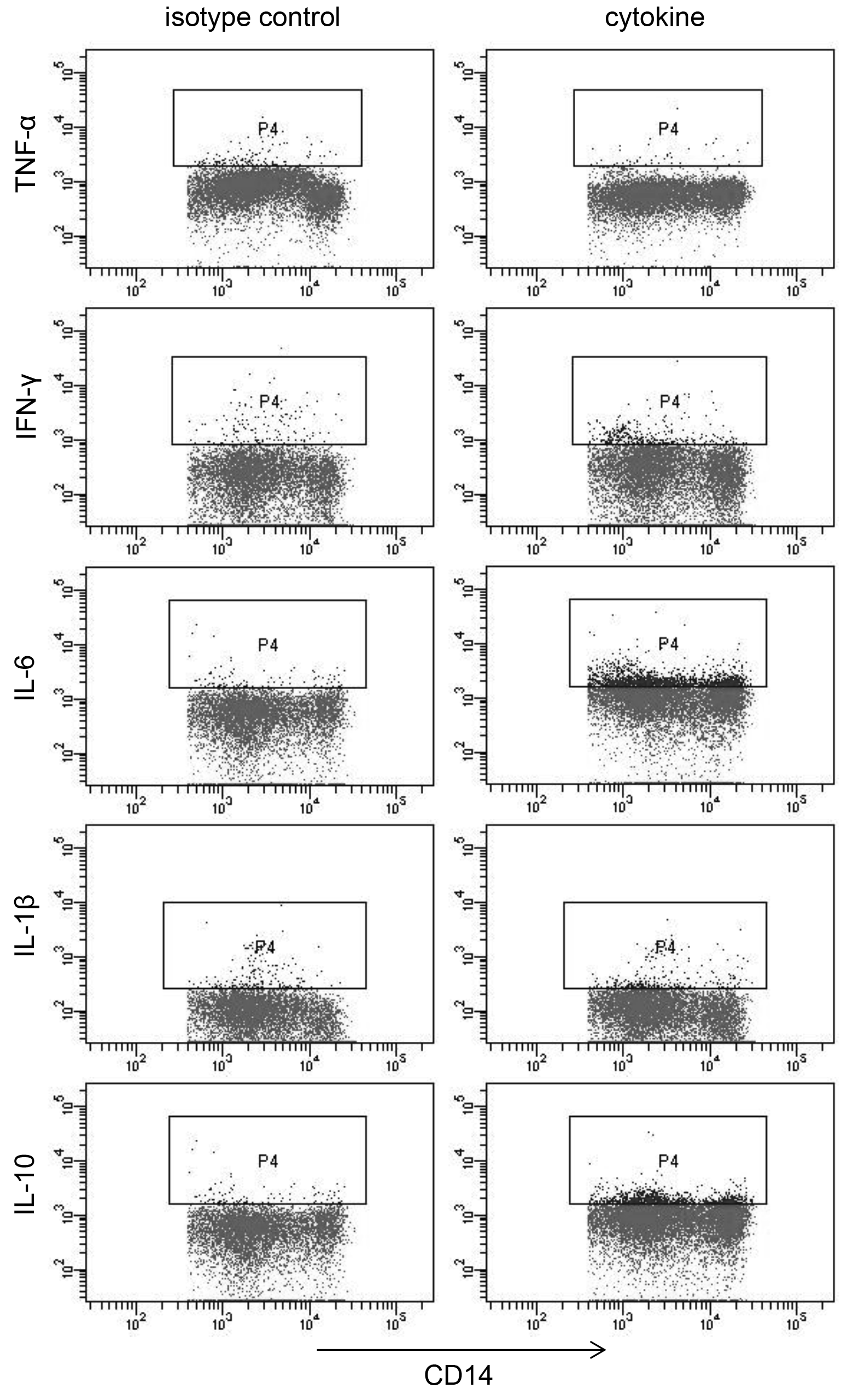

Supplement: Figure S6 — Isotype controls in unstimulated cells obtained from kidney transplant recipients. Representative FACS plots are shown of the isotype controls for the intracellular cytokine staining. Isotype controls in unstimulated cells for TNF-α, IFN-γ, IL-6, IL-1β and IL-10 (left side) and cytokine staining in unstimulated cells for TNF-α, IFN-γ, IL-6, IL-1β and IL-10 (right side). (TIF) [file pone.0070152.s006.tif]

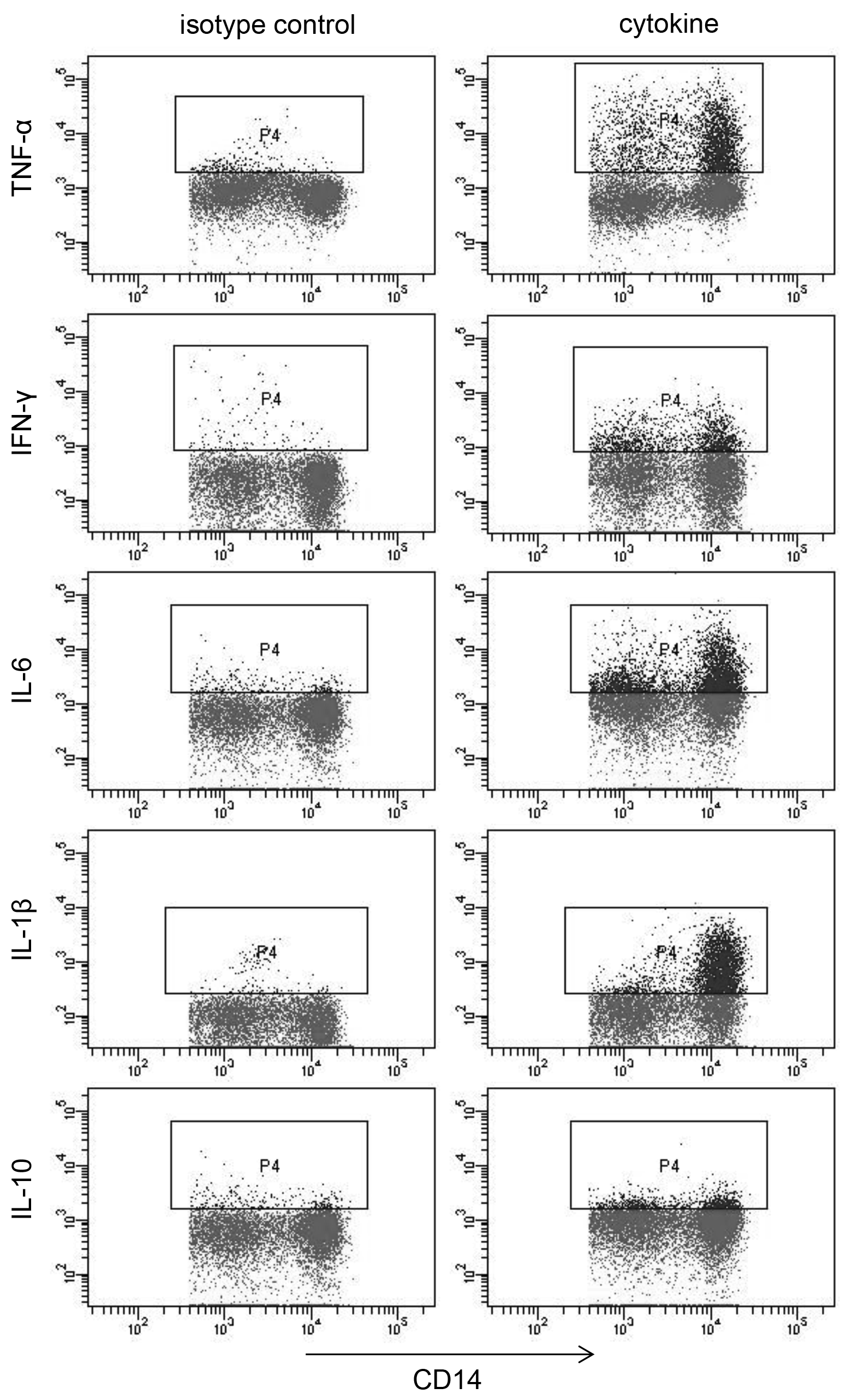

Supplement: Figure S7 — Isotype controls in IFN-γ and LPS stimulated cells obtained from kidney transplant recipients. Representative FACS plots are shown of the isotype controls for the intracellular cytokine staining. Isotype controls in IFN-γ and LPS stimulated cells for TNF-α, IFN-γ, IL-6, IL-1β and IL-10 (left side) and cytokine staining in IFN-γ and LPS stimulated cells for TNF-α, IFN-γ, IL-6, IL-1β and IL-10 (right side). (TIF) [file pone.0070152.s007.tif]

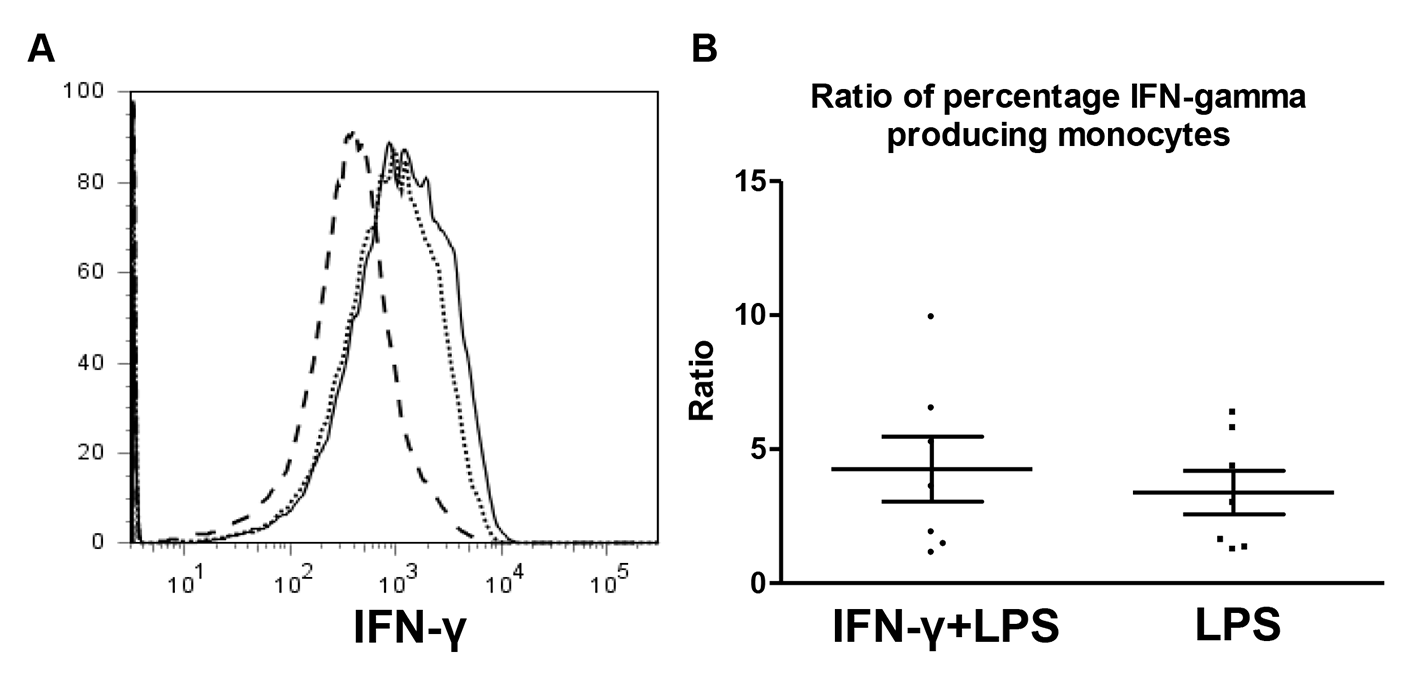

Supplement: Figure S8 — Percentage of IFN-γ producing monocytes after stimulation with either IFN-γ and LPS or LPS alone. Production of IFN-γ was tested after stimulation of freshly isolated PBMCs from recipients at time of Tx with either the combination of IFN-γ and LPS or LPS alone in the presence of golgiplug. The monocyte population was determined based on forward/sideward scatter, lack of expression of CD3, CD20 and CD56 and expression of CD14 and CD16. (A) Histogram of IFN-γ production of unstimulated monocytes (dashed line), IFN-γ and LPS stimulated (solid line) and LPS stimulated (dotted line). (B) The ratio of the percentage of IFN-γ producing monocytes with IFN-γ and LPS or LPS stimulation alone compared to the unstimulated situation was comparable. (TIF) [file pone.0070152.s008.tif]
